# Conceptual Considerations for Understanding Resilience in Healthcare Students

**DOI:** 10.1002/nop2.70061

**Published:** 2024-10-20

**Authors:** Claire Donnellan, Deepthi Chakkittakandy, Christina Lydon

**Affiliations:** ^1^ School of Nursing and Midwifery Edith Cowan University Joondalup Western Australia Australia; ^2^ School of Nursing and Midwifery, Trinity College University of Dublin Dublin Ireland; ^3^ Department of Practice Development Tallaght University Hospital Dublin Ireland; ^4^ School of Nursing and Midwifery University of Galway Galway Ireland

**Keywords:** healthcare students, healthcare systems, individual factors, protective factors, psychological well‐being, resilience, stressors

## Abstract

**Aims:**

Increasing attention has been given to the concept resilience in the context of healthcare especially during and post the COVID pandemic. Much of the inquiry and evidence reported has focused on promoting or enhancing resilience in healthcare for improving the quality of care and reducing medical negligence. This discursive paper aims to highlight how resilience is conceptualised and identify any potential limitations or gaps in the context of healthcare students, acknowledging considerations for further development and research into this topic.

**Design and Methods:**

This discursive discussion draws on relevant theoretical underpinnings from the fields of adjustment and coping psychology, and research and evidence from health sciences, for facilitating an understanding of resilience in supporting healthcare students to adapt into professional practice.

**Results:**

Investigation of resilience in healthcare students is mainly identified at an individual level as personal traits or skills for working within complex healthcare systems and clinical environments. Less attention has been given to examining resilience at the organisation or systemic level. This is primarily because of limited frameworks for investigating resilience from a multidimensional perspective recognising a wider systemic level influenced by external factors including socioecological determinants, for example, available support services for healthcare students.

**Conclusions:**

The link between resilience and its function to mitigate against associated neuropsychological distress and subsequent pathopsychological disorders in healthcare student cohorts is recognised; however, greater understanding of resilience as a multidimensional construct is warranted.

**Relevance to Clinical Practice:**

A multidimensional investigation of resilience is critical for the preparation and readiness of healthcare structures and organisations in facilitating the needs of healthcare students entering challenging and diverse healthcare working environments.

**Patient or Public Contribution:**

No patient or public contribution.

## 1 Introduction

The concept resilience has gained considerable momentum and increasing popularity regarding healthcare professionals and healthcare systems in response to the recent COVID global health pandemic (Gröschke et al. 2022; Rieckert et al. 2021). Resilience is seen as an important construct for healthcare professionals to have and is deemed necessary to maintain an effective, adaptable and sustainable workforce, important for employee well‐being and performance (Robertson et al. 2016). Developing resilient healthcare professionals has been associated with quality care and patient safety, where resilience can mitigate staff burnout, and in turn, prevention of risks associated with worse patient outcomes (Brigham et al. 2018). In relation to healthcare student cohorts training to become health professionals, the focus has tended to reflect on psychological health in terms of student readiness and preparedness for working within healthcare systems and environments (Mahroon et al. 2018; Walsh et al. 2010). However, the emphasis is now shifting more towards promoting and fostering resilience among health professional trainees (Kunzler et al. 2020; Wald 2020) with a view to creating resilience in health care (Aase et al. 2020) and resilient healthcare systems (Anderson et al. 2020). For the purposes of this discursive paper, healthcare students refer to students completing a programme of study in health sciences and in training for a health profession to include nursing, medicine, dentistry, paramedics, pharmacy, physical and physiotherapy, occupational therapy, dietetics, speech and language therapy, social work, chiropody, podiatry and psychology (World Health Organization 2013). The increasing attention given to the concept resilience in the context of healthcare professionals is evident in the exponential increase in articles published on the topic with particular emphasis being given to healthcare students within the last 15 years. Prior to this, the focus had been on students' response and adaptation to stress and their overall health and well‐being and less emphasis on building hardiness or resilience in students in preparation for a career in healthcare. The aim of this article was to discuss how resilience is conceptualised in the context of healthcare students, identify any potential limitations or gaps in its understanding and acknowledge considerations for further development and research into this topic. More specifically, we will discuss an overview regarding its definitions, frameworks used to examine resilience in healthcare, its measurement and assessment, factors associated with resilience, and any known strategies or interventions used to enhance resilience in the theoretical and clinical training of healthcare students.

## 2 Defining and Understanding Resilience as a Concept

Resilience has generally been conceived as the capacity to recover or bounce back from extremes of trauma, deprivation, threat or stress or return to a previous state after a time of stressful transition or an adverse event (Atkinson, Martin, and Rankin 2009; Southwick and Charney 2012). The term resilience appears to have been first used and defined by researchers predominantly working in child psychology and family therapy and refers to a *dynamic process encompassing positive adaptation within the context of significant adversity* with two critical conditions: (1) exposure to significant threat or severe adversity and (2) the achievement of positive adaptation despite major assaults on the developmental process (Luthar, Cicchetti, and Becker 2000). The American Psychological Association (2018) concur with this definition indicating resilience as the process and outcome of successfully adapting to difficult or challenging life experiences, especially through mental, emotional and behavioural flexibility and adjustment to external and internal demands (American Psychological Association 2018). Factors that contribute to how well individuals adapt to adversities include ways in which they view and engage with the world, the availability and quality of social resources and use of specific coping strategies (Donnellan et al. 2006). There is an onus on higher education institutions to be providers of social resources required and training healthcare students in using adaptative coping strategies.

It has been conceptualised as an individual characteristic, viewed as a personal or fixed trait and as a measure of stress coping ability (Connor and Davidson 2003). Others define it as the capacity of an individual to avoid negative social, psychological and biological consequences of extreme stress that would otherwise compromise their psychological or physical well‐being (Russo et al. 2012) or it is viewed as the maintenance of positive adaptation by individuals despite experiences of significant adversity (Luthar, Cicchetti, and Becker 2000). These conceptualisations are in keeping with resilience theory presented as three waves of resiliency inquiry. The first wave is the identification of resilient qualities characterised through phenomenological identification of developmental assets and protective factors, the second wave describes resilience as a disruptive and reintegrative process for accessing resilient qualities and the third wave exemplified the postmodern and multidisciplinary view of resilience, which is the force that drives a person to grow through adversity and disruptions (Den Hartigh and Hill 2022; Richardson 2002). More recently, the concept has also been viewed as the intrinsic ability of a system to adjust its functioning in response to changes, sustaining operations even during disturbances, mishaps and stressful events (Nemeth et al. 2008). Despite the consequences of adapting less positively to adverse situations and environments leading to potential psychological disorders (Southwick and Charney 2012), the concept remains vague in relation to health professions because of diversity in definition and measurement (Huey and Palaganas 2020). An even newer conceptualisation or paradigm known as resilience engineering has evolved over the last decade (Anderson et al. 2016; Nemeth et al. 2008) and encompasses individual, organisational and systemic factors leading to quality and safety across healthcare systems. Resilience engineering has shifted the focus for the healthcare industry preventing incidents and errors and reactively responding to negative outcomes in health systems to proactively learn and adapt at multiple levels (Goel 2022). This approach enables a wider focus that expands the field of resilience investigation beyond how individuals are resilient in being able to adapt to all elements of threat and adversity within healthcare environments but to also consider how healthcare systems are fit for purpose for individuals to operate within.

Another important distinction to highlight is that resilience can be interpreted as having both a psychological and physiological basis. This is particularly useful for having a clearer understanding of resilience at an individual level, where emphasis is placed on the neurobiological basis of resilience and its association with mental health and the predisposition of psychological disorders among university students in general (Auerbach et al. 2018). Resilience reflects the absence of psychological distress or disorders following exposure to adverse situations, trauma or perceived traumatic events. Therefore, it may also be considered in part as either controlling the perceived appraisal of the stressful event or the response to stress or minimising the effect of stress in the face of adversity. It is generally well accepted that the hypothalamic–pituitary–adrenal axis (HPA) and the sympathetic nervous system (SNS) are dysregulated in response to stress directly at the level of the hypothalamus or indirectly at the level of the amygdala (aan het Rot, Mathew, and Charney 2009). The duration of exposure to stressful events and the stressor intensity determines the regulation of HPA axis and the SNS in response to stress (Herman et al. 2016). Hyperactivity of the HPA axis occurs because of increased exposure to stress that in turn, correlates with increased risk for major depressive disorder (MDD) (Southwick and Charney 2012). Early recognition of healthcare students' responses in new clinical environments in terms of their resilience levels is significant in these cohorts' initial training and limiting duration of exposure to healthcare placements perceived as highly stressful when levels of resilience are low.

## 3 Frameworks for Consideration When Examining Resilience in Healthcare Students in the Clinical Environment

Current frameworks for examining resilience in healthcare professionals and more specific to healthcare students, addressing individual and organisational levels collectively are scarce. However, there are existing frameworks in the wider psychology literature examining resilience that may facilitate investigation in healthcare students. It is important to view resilience as a multidimensional construct at the individual, and organisational or systemic levels as illustrated above, when understanding the concept from the perspective of healthcare students navigating and engaging in diverse and adverse healthcare environments. Aforementioned, the individual level includes the internal process of adapting well in the face of adversity to significant sources of stress or threats influenced by biological, psychological, social and cultural factors that interact with one another to determine how one responds to stressful experiences (Southwick et al. 2014). At the organisational or systemic level, resilience is also interpreted as adapting well in the face of adversity or to bounce back during a crisis or significant event and is interpreted as an outcome as in when organisations perform well (Duchek 2020). Understanding resilience at the organisational compared to the individual level is deemed a more complex and broader construct with various interpretations that include anticipation, adjustment, resistance and recovery (Duchek 2020).

Given the lack of consensus in determining the various facets of resilience, a framework called the Multi‐System Model of Resilience (MSMR) has been proposed that captures intraindividual, interpersonal and socioecological factors that contribute to the interactive process of resilience that is dynamic and multidimensional (Liu, Reed, and Girard 2017). A newer proposed version includes three systems within the MSMR to include internal resilience (sources of resilience within the individual, including health and health‐related factors that promote well‐being), coping and pursuits (coping‐related skills, knowledge and goals that allow the individual to respond to challenges and needs) and external resilience (socioecological factors that promote resilience, such as access to healthcare and support services) (Liu, Reed, and Fung 2020). This framework was applied to examine resilience in psychology students and findings collectively provided a strong theoretical rationale for the three‐system structure, highlighting the importance of tapping into the multidimensionality of resilience (Liu, Reed, and Fung 2020). This framework shows promise and underscores the importance of future investigations and refinements for use in other healthcare student cohorts.

Resilience engineering, a subfield of safety science research focusing on understanding complex adaptive systems cope when encountering unanticipated events, has led to the development of structuring frameworks that address resilience as a concept at the organisation and systemic levels of health care through the deliberate design and construction of systems that have the capacity of resilience (Fairbanks et al. 2014). One such framework that has been developed is the concepts for applying resilience engineering (CARE) model (Anderson et al. 2016). Anderson et al. (2020) argue for further theoretical development in resilience research to include all levels and scales of activity across the whole healthcare system based on what the authors call the Integrated Resilience Attributes Framework (Anderson et al. 2020). This framework expands to encompass the CARE model and moments of resilience model, to recognise the prominence of social, cultural and organisational factors in healthcare work. The framework provides guidance for researching resilience, more specifically for mapping processes of anticipating, responding, monitoring and learning at different scales of time and space across the entire healthcare system. Although frameworks employing resilience engineering principles may play a significant role in the quality and safety assurances of healthcare systems, they require the learner to be at an advanced stage in their apprenticeship with substantial technical ability, knowledge and experience (Fairbanks et al. 2014), to anticipate the right course of action to avoid failures and lead to successful outcomes at individual, team and organisational levels. The suitability of these types of frameworks exploring the wider concept of resilience in healthcare systems may not be applicable to health professionals in training in undergraduate programmes of study. However, such approaches may well be appropriate for understanding health professionals operating within healthcare systems and environments during advanced training as in graduate programmes of study and beyond.

## 4 Measuring and Assessing Resilience in Healthcare Students

The diverse conceptualisation of resilience has also resulted in a broad range of measures used to assess resilience for specific cohorts across the lifespan to include assessment in children and adolescents where the concept was first devised, in military personnel where psychological harm is high risk in warzone regions, and in more recent years in healthcare professionals working in highly stressful clinical environments (Britt et al. 2016; Rodman et al. 2019). The focus of assessment has generally been on establishing individual personality traits or internal factors. Some of the commonly used generic assessment measures include the Brief Resilient Coping Scale (BRCS) (Sinclair and Wallston 2004), Brief Resilience Scale (BRS) (Smith et al. 2008), Conor‐Davidson Resilience Scale (CD‐RISC) (Connor and Davidson 2003) and the Dispositional Resilience Scale (DRS) (Bartone et al. 1989) that have been used to assess resilience in healthcare professionals and healthcare student cohorts. To date, there are no established measures of resilience devised exclusively for use in health professional cohorts resulting in studies examining resilience in healthcare professional cohorts using the well‐established generic measures.

Other available measures have focused on assessing protective factors for resilience, for example, Baruth protective factors inventory (BPFI) (Baruth and Caroll 2002), protective factors for resilience scale (PFRS) (Harms, Pooley, and Cohen 2017) and scale of protective factors (SPF) (Ponce‐Garcia, Madewell, and Kennison 2015) as indicating a demonstration of resilience. Resilience has also been captured in measures of outcome or functional indicators that would typically include assessment of the challenge, trauma or adversity and consequential psychological responses or functioning, that is, absence of psychological distress or dysfunction in the face of adversity. The focus of resilience assessment in healthcare students has tended to reflect either their internal traits or protective factors as considered capacity for resilience rather than functional indicators that assess the actual challenges or adversities students experience in healthcare environments. However, their psychological responses to challenges or adversities such as course work pressures or highly stressful clinical placements (Henderson, Chetty, and Gurayah 2021), have been assessed from a psychological or mental health perspective in the form of psychological outcomes such as levels of depression, anxiety and coping strategies (Kotera et al. 2021). These assessments would represent functional indicators of resilience as psychological functioning with less emphasis on the actual adversities, trauma experienced or challenges faced by healthcare students. A comprehensive systematic review examining interventions to foster resilience in healthcare student cohorts reported that the CD‐RISC was the most used scale in six out of 17 studies, followed by the resilience subscale of psychological capital questionnaire (PCQ) (Kunzler et al. 2020). A range of other assessments and scales are often used to predict or be associated with resilience, for example, satisfaction of competence (Neufeld and Malin 2019); life satisfaction (Kim 2019); empathy (Chan et al. 2020; Mathad, Pradhan, and Rajesh 2017); well‐being (Labrague 2021); perseverative thinking (Mathad, Pradhan, and Rajesh 2017) and personality type (Avrech Bar et al. 2018; Carter et al. 2016; Škodová and Bánovčinová 2018).

## 5 Factors Associated With Resilience in Healthcare Students

The evidence to date examining factors associated with resilience in relation to healthcare students is also reflective of investigation at the individual or internal factors level and not identifying associated factors at the organisational or systemic levels. Different factors or variables are considered in association with resilience in terms of correlating with it as a construct, as surrogate traits of resilience, as protective factors of resilience or as outcomes to demonstrate resilience. Factors correlating with resilience as a construct have included use of coping strategies or a method of adaptation or adjustment (Farquhar, Kamei, and Vidyarthi 2018; Sanderson and Brewer 2017; Thompson et al. 2016). Coping or use of coping strategies are often described in tandem with resilience although the distinction between coping and resilience is not always explained. A recent cross‐sectional study has reported coping and resilience to be distinct, but are related constructs and are likely to influence each other (Van der Hallen, Jongerling, and Godor 2020). One study in student nurses found coping strategies to have an intermediary function between resilience and clinical communication ability (Li, Hua, and Yang 2021). Another study also examined resilience in students nurses, reported high trait (to include prosocial, cognitive, interpersonal, and school functioning factors), state (to include self‐regulation and self‐capacity) and combined resilience using prevalidated scales (Alkaissi et al. 2023). Surrogate traits of or defined as part of the concept resilience in healthcare student studies include self‐efficacy, reflective ability and self‐confidence (Walsh et al. 2020). Protective factors of resilience include self‐efficacy, optimism, emotional intelligence and self‐stewardship/self‐care (Hughes et al. 2021). Outcomes to demonstrate resilience involve alleviating student academic burnout, psychological well‐being, academic performance (Wei et al. 2021), anxiety, depression, stress and perceived stress, well‐being and quality of life (Kunzler et al. 2020). Identifying factors such as cognitive functioning, emotional regulation, social skills, physical health and neurobiological functioning can determine either appropriate therapeutic interventions or resilience protective factors to mitigate against psychological disorders in response to stress, stress‐related events and environmental stressors. There is an ongoing argument in the literature on whether resilience is identified as a personality trait or placing it in the class of skills. The evidence to support resilience as a personality trait is dependent on whether other personality traits are assessed in similar studies and reported to correlate with each other in a similar direction and if there are longitudinal data to report resilience remaining stable over time. The arguments in favour of resilience to be part of a class of skills tend to be supported in interventions studies aiming to enhance or improve resilience or that it is somehow reinforced through therapeutic approaches (Leys et al. 2020).

## 6 Interventions to Enhance Resilience in Healthcare Students

The purpose of resilience interventions have been considered to prevent or offset against mental health‐related issues and psychological disorders in higher education student cohorts (Ang et al. 2022). There has been greater attention given to efforts determining actual interventions that enhance or promote resilience in healthcare students in comparison to distinguishing specific factors associated with resilience. The emphasis is on building, developing and promoting resilience in healthcare student cohorts (Chan et al. 2020; Lopez et al. 2018; Thomas and Asselin 2018; Walsh et al. 2020) although a more in‐depth understanding of resilience is still lacking. A recent Cochrane review that included 30 randomised controlled trials reported very‐low certainty evidence for the effect of resilience training on resilience, anxiety and stress or stress perception and no evidence of any effect on depression or well‐being or quality of life (Kunzler et al. 2020). Resilience training interventions reported in that review included training methods that addressed resilience factors to include meaning in life or purpose in life, sense of coherence, positive emotions or positive affect, hardiness, self‐esteem, active coping, self‐efficacy, optimism or positive attributional style, social support, cognitive flexibility and religious coping (Kunzler et al. 2020). A more recent systematic review reported on the use of educational strategies reflection, positive reframing, problem‐based learning and mindfulness to enhance student nurses' internal protective factors (Hughes et al. 2021). Advice written for pharmacy students included to take strategies to prevent burnout and to move towards a state of resilience and well‐being (Phillips, Bekelian, and Billett 2020). These practical steps involved making students aware of all available resources to them on and off campus including mental health screening and counselling services, seeking help early, developing social supports, finding meaning in work, strive for balance and practice healthy lifestyle habits. An argument that has been made in relation to adverse events or trauma experienced in childhood and adolescent cohorts, supports evidence of cognitive reappraisal training for better emotional regulation to offset the negative impact of such events (Rodman et al. 2019). In university students in the United Kingdom, one study reported mindfulness mediated the relationship between cognitive reappraisal and psychological resilience and may need to be considered for improving academic performance and psychological well‐being (Zarotti, Povah, and Simpson 2020). Another intervention study reported using a multidimensional stress prevention program, that integrated mindfulness‐based activities, cognitive and behavioural strategies, social skills and emotional regulation exercises (Recabarren et al. 2019), but no specific studies have been reported to examine the relationship between cognitive appraisal or training to improve positive appraisal exclusively in healthcare students.

## 7 Limitations and Possible Gaps

The nature of resilience as a construct or concept is not fully understood in the context of healthcare students either from a theoretical or methodological perspective that includes a consistent approach to assessment or operationalisation across the different healthcare student groups. There have been strives towards developing interventions that build and promote resilience in healthcare students (Kunzler et al. 2020) without fully determining the factors and mechanisms that represent resilience in these student groups. For working in high stress‐related healthcare settings such as hyperacute or critical care units, having resilient or resilient like attributes or characteristics is perceived very positively for either protecting against or managing psychological consequences from adverse clinical experiences in these environments. However, the detrimental consequences for healthcare students from exposure to adverse environments especially in their early apprenticeship years, can result in psychological distress for prolonged periods leading to psychopathology to include anxiety and depression (Mahroon et al. 2018) and in some cases posttraumatic stress disorder risk symptoms (Lee et al. 2021). Therefore, interventions that mitigate against such consequences need to focus on training in cognitive reappraisal, coping self‐efficacy and managing emotion regulation (Southwick and Charney 2012). Efforts to address these potential consequences of psychological distress for healthcare students may require inclusion of cognitive, behavioural and social factors, emotional regulation, physical health and any neurobiological risk factors for an overall comprehensive student health assessment regarding their welfare and well‐being for functioning in clinical training environments.

The exponential increase in publications addressing resilience in healthcare students in the last 15 years and even more recently again due to the COVID pandemic demonstrates greater demands are now placed on healthcare students. This emphasises the importance of the concept resilience in relation to healthcare students' mental health and how this in turn may impact workforce levels in healthcare and health professionals' readiness for clinical practice. The greatest focus is on examining resilience in nursing and medical students because these cohorts make up the largest proportion of healthcare students in the healthcare sector and are directly positioned in healthcare provision compared to other allied healthcare and health‐supporting services student cohorts. We have aimed to identify gaps and limitations regarding the investigation of resilience in healthcare students throughout each of the sections above to include inconsistency in defining resilience; a scarcity in frameworks to examine resilience that are specific to healthcare students and a broad range of measures available to assess resilience, developed in other cohorts such as children and military professionals. The inconsistency in the conceptual definition of resilience is not exclusive only to healthcare student studies because the diversity tends to exist across the wider generic literatures including in developmental and organisational psychology (Duchek 2020; Mayor‐Silva et al. 2021) and therefore this is not unique for its use in the healthcare literature. We do acknowledge that frameworks specific to healthcare students are scarce and have introduced possible frameworks for consideration which may serve very well for this cohort of students. We have identified in all sections where areas or available information may be limited to some extent and emphasise our intent is to bring the broader approaches of resilience mainly used in psychology literature, to a health professional audience for possible consideration and use.

## 8 Future Research Considerations and Conclusion

The relationship between resilience and other variables that may be risk factors for psychological distress or are therapeutic interventions such as mindfulness, self‐efficacy, active coping, emotional intelligence, psychological well‐being and burnout requires further research examination to determine which variables are measures of resilience traits, protective factors for resilience, functional indicators of resilience or outcomes post resilience interventions including those assessing psychological well‐being. There are specific trends not fully explored regarding how resilience impacts or influences other variables of interest as in whether it acts as a mediator or moderator between stress and psychological well‐being and between burnout and communication in healthcare student cohorts. The evidence to date indicates resilience as a predictor or having a strong association with mental health status and psychological well‐being of student cohorts working in highly stressed environments such as healthcare settings. However, further investigation is required in the field of psychological health and well‐being of healthcare students to determine how resilience is defined and to identify resilience at the individual level and as a multidimensional construct that is inclusive of factors influencing at the organisational and systemic levels. There would be merit in comparing resilience across the different healthcare student groups to examine if there are similar trends or differences in relation to how various disciplines (e.g. nursing, medicine, pharmacy and other healthcare student groups) cope, manage and adapt in healthcare clinical environments and settings. Future studies and reviews may also consider examining the differences in training and clinical healthcare working environments across the various disciplines.

In conclusion, understanding resilience in healthcare students is mainly conceptualised in relation to individual and personal factors without the wider socioecological perspective being taken into consideration. Efforts are being made from a research intervention perspective to promote resilience in healthcare students in the absence of additional attention and emphasis given to enhancing resilience across the wider healthcare provision systems and associated educational institutions. There is an urgent need for the use of theoretical models and frameworks that recognise and include social, cultural and organisational factors for investigating resilient performance in healthcare work (Anderson et al. 2020) which is of particular importance in the understanding and management of psychological health of healthcare students. Further research investigation is required to examine all relevant factors that may mediate or moderate the relationship between resilience and potential mental health vulnerability in healthcare students. Therefore, a detailed review on factors associated with resilience is required for understanding broader psychological individual aspects and adaptation to the external healthcare clinical environment, thereby adding great advantage to the field of healthcare student well‐being.

## Author Contributions

All authors assisted with conceptualising and designing the article. C.D. wrote the original draft of the manuscript and all authors revised it and have given final approval to the version to be published.

## Ethics Statement

The authors have nothing to report.

## Consent

The authors have nothing to report.

## Conflicts of Interest

The authors declare no conflicts of interest.

## Data Availability

This is a discursive paper of published literature. References of articles used in this evidence synthesis are provided throughout the manuscript. The papers analysed are available to the public.
